# Land use change and rodenticide exposure trump climate change as the biggest stressors to San Joaquin kit fox

**DOI:** 10.1371/journal.pone.0214297

**Published:** 2019-06-12

**Authors:** Theresa Nogeire-McRae, Joshua J. Lawler, Nathan H. Schumaker, Brian L. Cypher, Scott E. Phillips

**Affiliations:** 1 Conservation Science Partners, Corvallis, Oregon, United States of America; 2 School of Environmental and Forest Sciences, University of Washington, Seattle, Washington, United States of America; 3 Department of Fisheries and Wildlife, Oregon State University, Corvallis, Oregon, United States of America; 4 California State University Stanislaus, Endangered Species Recovery Program, Turlock, California, United States of America; Smithsonian Conservation Biology Institute, UNITED STATES

## Abstract

Animal and plant species often face multiple threats simultaneously. We explored the relative impact of three major threats on populations of the endangered San Joaquin kit fox. This species was once widely distributed across the southern San Joaquin Valley, California, USA, but agriculture and urban development have replaced much of its natural habitat. We modeled impacts of climate change, land-use change, and rodenticide exposure on kit fox populations using a spatially explicit, individual-based population model from 2000 to 2050 for the Central Valley, California. Our study indicates that land-use change will likely have the largest impact on kit fox populations. Land development has the potential to decrease populations by approximately 15% under a compact growth scenario in which projected population increases are accommodated within existing urban areas, and 17% under a business-as-usual scenario in which future population growth increases the developed area around urban centers. Plausible scenarios for exposure to pesticides suggest a reduction in kit fox populations by approximately 13%. By contrast, climate change has the potential to ameliorate some of these impacts. Climate-change induced vegetation shifts have the potential to increase total available kit fox habitat and could drive population increases of up to 7%. These vegetation shifts could also reduce movement barriers and create opportunities for hybridization between the endangered San Joaquin kit fox and the more widely distributed desert kit fox, found in the Mojave Desert. In contrast to these beneficial impacts, increasing climate extremes raise the probability of the kit fox population dropping below critical levels. Taken together, these results paint a complex picture of how an at-risk species is likely to respond to multiple threats.

## Introduction

Most species threatened with extinction face multiple, often interacting, threats [1,2]. For many of these species, the loss or fragmentation of habitat is the leading threat, and conversion to agriculture is the most common reason for habitat loss [1,3]. However, without significant, successful, and swift efforts to reduce greenhouse-gas emissions, climate change may soon rival land-use change as the leading threat to many species and ecological systems [3].

Land-use change can affect species in multiple ways, primarily through the direct consequences of habitat loss and the more difficult to quantify indirect effects associated with fragmentation. For example, animals using habitat in and around agriculture and low-density developed areas are increasingly prone to human-based threats such as vehicle strikes when crossing roads, competition from synanthropic species, and exposure to pesticides such as second-generation anticoagulant rodenticides [4].

Climate change is already altering species distributions, phenologies, interspecific interactions, the composition of communities, and ecosystem functions [5]. In California’s San Joaquin Valley, temperatures are projected to be warmer in both winter and summer, with a mean annual increase of 1.4–2.0°C by 2070 [6]. Also, precipitation is projected to decrease 9% to 30%, albeit with a high degree of uncertainty in magnitude [6]. Periods of extreme high summer temperatures are projected to become longer, hotter, and more frequent, and the resulting droughts more severe [7].

The San Joaquin kit fox (*Vulpes macrotis mutica*, “kit fox”) is an endangered subspecies of the more widely occurring kit fox. These kit foxes live in the arid grass- and shrub-lands around the perimeter of the San Joaquin Valley in central California, where they preferably hunt kangaroo rats, but also consume ground squirrels, rats, mice, birds, and insects. The current population size of the kit fox is unknown, but is estimated to be fewer than 4200 individuals [4,8].

Since the mid-1800s, at least 70% of the land in the kit fox’s range has been converted to irrigated agricultural, and to a lesser extent, urban and industrial uses [9]. The resulting habitat loss was a primary factor in the 1967 listing of the subspecies as endangered under the U.S. Endangered Species Act [10,11]. Other significant threats to the species include the agricultural industry (through its extensive use of rodenticides) [4], the solar-power industry (which is expanding into kit fox habitats), and climate change, which changes prey availability and alters habitat quality (because kit foxes cannot use dense vegetation, more arid conditions could improve kit fox habitat by reducing grass and shrub cover).

Here, we assessed the effects of land-use change, climate change, or exposure to rodenticides on kit fox populations, across their range, using a spatially-explicit individual-based model. We estimated current population size for the fox from 2010–2060 and assessed how this number might change under simulated future disturbance scenarios. In addition to current conditions, we modeled two land-use change scenarios and three climate-change scenarios, as well as the impacts of rodenticide exposure.

## Methods

**Fig 1:**
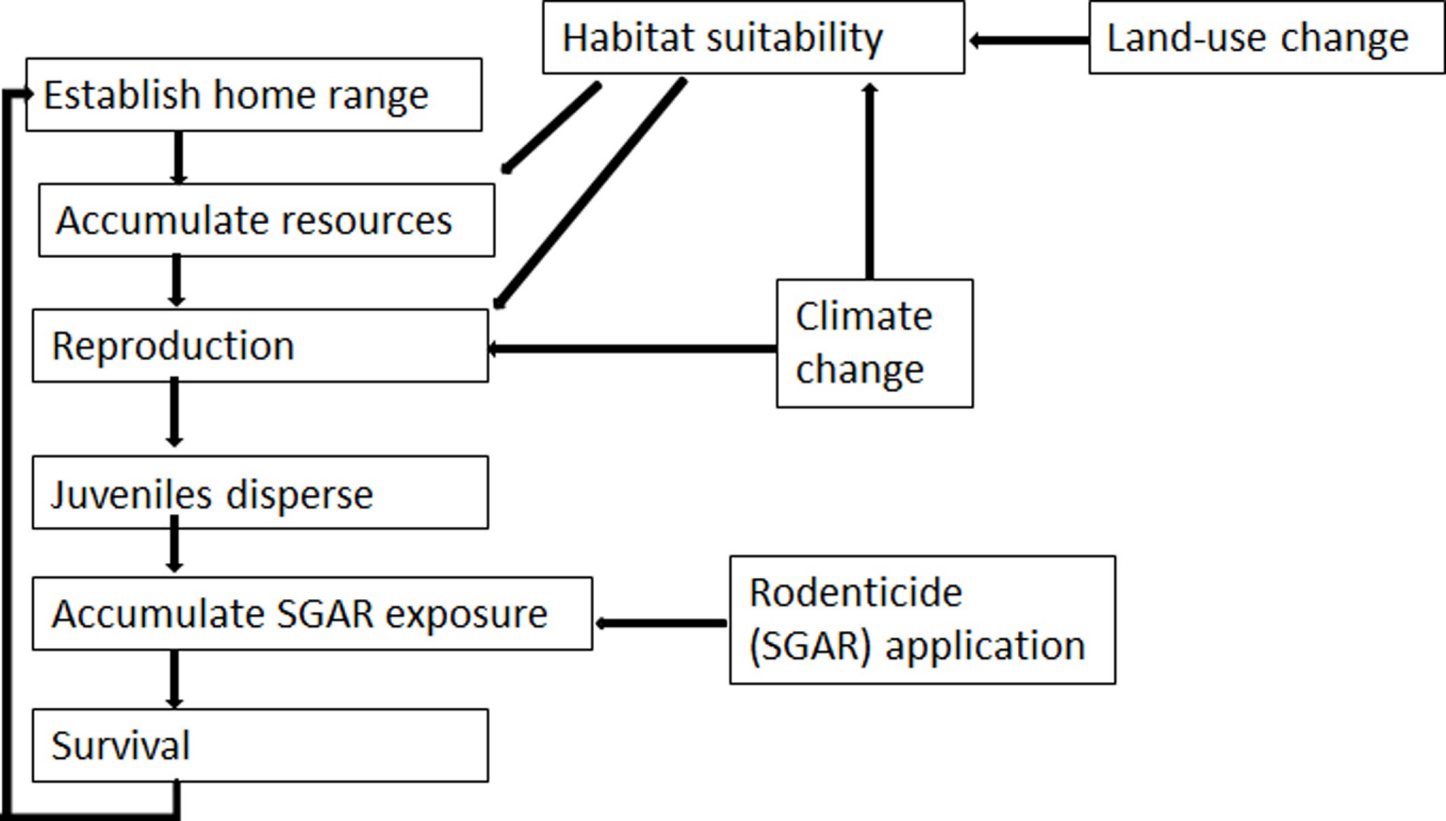
Model schema. Land-use change and climate change affect kit fox populations via two pathways: by influencing the quality of the “resources” (food, shelter) available to foxes, and by impacting reproductive success. Rodenticide exposure, meanwhile, influences populations via survival rates.

We used the spatially explicit, individual-based population modeling platform HexSim [12] to simulate the impacts on kit fox of climate change, land-use change, and rodenticide exposure (Fig 1). We used HexSim to simulate individual kit foxes across 81,246 km^2^. Our HexSim model simulated the kit fox life history, including reproduction, dispersal, prospecting, resource acquisition, exposure to rodenticides, and survival. The model made use of a habitat suitability map [4,8] to simulate dispersal, prospecting, and resource acquisition. Our model integrated life history traits, repeated exposures to rodenticides, and spatial data layers describing habitat and locations of likely exposures. We modeled female kit foxes using yearly time steps in which each individual had the potential to disperse, establish a home range, acquire resources from their habitat, reproduce, accumulate rodenticide exposures, and die. Simulated kit foxes established home ranges with sizes that were inversely proportional to habitat quality. Both survival and reproductive were influenced by resource allocation, with resource-poor individuals experiencing lower vital rates. Density dependence emerged as a consequence of the competition between individuals for finite available resources. The probability that an individual was exposed to rodenticides varied based on land cover class [4]. We base our findings here on 100 replicate simulations run for each model scenario.

### Land-use change scenarios

The human population of the San Joaquin Valley is projected to increase from 3.3 million in 2000 to nearly 8 million in 2050 [13]. We developed two scenarios based on detailed land-use change projections from 2000 to 2050 for the Central Valley, California (UPlan Urban Growth Model, [13]). The first, “status quo” scenario assumed no significant changes would be made to urban growth policies. The second, “compact growth” scenario modeled a policy in which the same population growth is accommodated by increasing densities rather than developing outside the existing urban population centers [13].

To build habitat suitability maps for both scenarios and for 10-year increments, we began with a baseline kit fox habitat suitability map [4], and then used projected land-use changes provided by UPlan to alter this map (see S1 Table). New suitability values were assigned using the same habitat suitability values as the original habitat suitability model. For the two classes not described by the habitat model (high density commercial and residential with 10-acre lots), we estimated intermediate values. The land-use change scenarios were developed for the year 2050, so we interpolated intermediate years at 10-year increments by randomly selecting 1/5 of the projected change for each of five 10-year periods. By convention, we always used the minimum habitat suitability value taken from the projected UPlan land-use map and the baseline map. We assumed that the rare increases in habitat suitability were due to mapping errors given that the scenarios included no abandonment of agricultural or developed land, but rather only added new development. We then used these new habitat suitability maps in our kit fox simulation model for the ensuing 10-year periods.

### Climate change induced vegetation change scenarios

We incorporated the effects of climate change in two ways. First, we considered the long-term effects of changes in precipitation and temperature on habitat structure. We used projected future potential vegetation from an empirical model linking broad vegetation types to climate [14]. We used model outputs based on climate-change projections from the Community Climate System Model (CCSM3.0) from the National Center for Atmospheric Research for the period from 2038–2069 and the Geophysical Fluid Dynamics Laboratory (GFDL) model for the period from 2038–2070. We then measured the change in kit fox habitat suitability between the modeled future climate and the modeled current climate and applied this difference to the baseline habitat suitability map and the modeled suitability for each time increment. The future vegetation maps were averages from approximately 2038–2069. We interpolated the change in 10-year increments from 2010–2040, and then used the average value from 2040 through the end of the simulation.

### Increased variability in reproduction due to climate change

We also considered the effects of climate change on kit fox reproduction. Models generally agree that extreme precipitation events are likely to become more common and intense in California [15,16], which in turn could cause more variability in kit fox population sizes. It is also expected that interannual variation in precipitation could lead to more consecutive dry days, which could negatively impact kit fox reproduction. The densities of the kit foxes’ preferred prey—herbivorous and granivorous rodents—are closely tied to primary productivity, and therefore, to rainfall. Kit fox populations are generally regulated by the availability of their prey and thus, population numbers tend to fluctuate with rainfall [17].

We simulated the potential effects of increasing extremes in precipitation on reproduction by varying reproductive rates in the model. We modeled the effect of increasing inter-annual variability in rainfall by increasing the variability of the foxes’ reproductive success. To simulate this in our model, we created “average,” “good” and “bad” year categories which subsequently influenced reproduction. In each time step, the quality of the year was chosen randomly with the following probabilities: good (1/5), bad (1/5), and average (3/5). Reproductive output (number of foxes born) in a good year had an expected value that was 80% greater than that of an average year.A bad year had an expected value that was 80% less than an average year. These reproductive rates and probabilities of good and bad years are based upon models of rainfall impact on kit fox [17] and kangaroo rat [18] populations.

### Rodenticide exposure effects scenarios

To simulate the likely impacts of rodenticide exposure on kit fox populations, we used a map of areas of likely exposure to rodenticides developed from pesticide-use data reported to the California Department of Pesticide Regulation [19], liver tests of kit foxes [20], and landowner surveys [21]. We used California agricultural databases, surveys of residents, and results from laboratory measurements to assemble a map quantifying the potential for rodenticide exposure [4]. Territorial kit foxes’ exposure was based on the placement and size of their home range. For non-territorial kit foxes, exposure was based on the areas that were visited in the process of prospecting for a territory. Newborn kit foxes were assigned the same exposure value as their mothers. For the purposes of influencing vital rates, kit foxes which experienced any exposure were placed into either "low", "medium", or "high" exposure bins, with the bin boundaries set to ensure that roughly 1/3 of the population fell into each exposure class. We imposed pesticide-induced mortality rates upon individuals that the model suggested would become exposed to rodenticides on the landscape. These exposure risks were accumulated throughout each season, resulting in an overall exposure for each annual time step. Here, we consider an intermediate exposure effect scenario wherein foxes that were exposed to rodenticides suffered an additional 2% - 25% mortality rate, depending on the amount of exposure.

## Results

**Fig 2:**
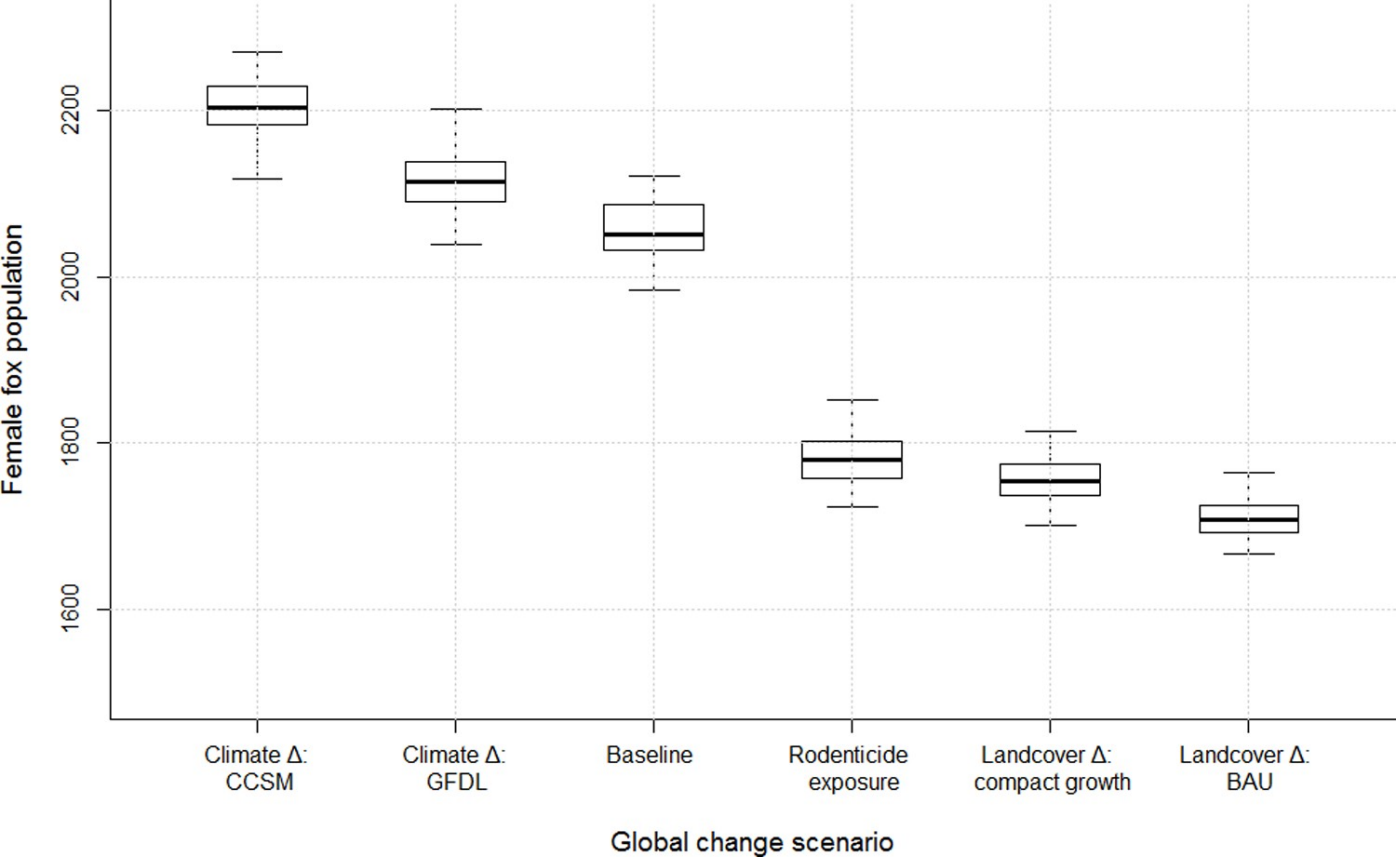
Modeled population size of female kit foxes under six scenarios. Both climate change scenarios resulted in larger modeled kit fox populations, while rodenticide exposure and two landcover change scenarios resulted in smaller modeled kit fox populations, relative to the baseline scenario.

Projected land-cover change caused the greatest negative impact to our simulated kit fox population (Fig 2). Exposure to rodenticides also had a negative effect, and projected climate-induced vegetation changes result in slightly positive effects.

### Impact of land use change

**Fig 3:**
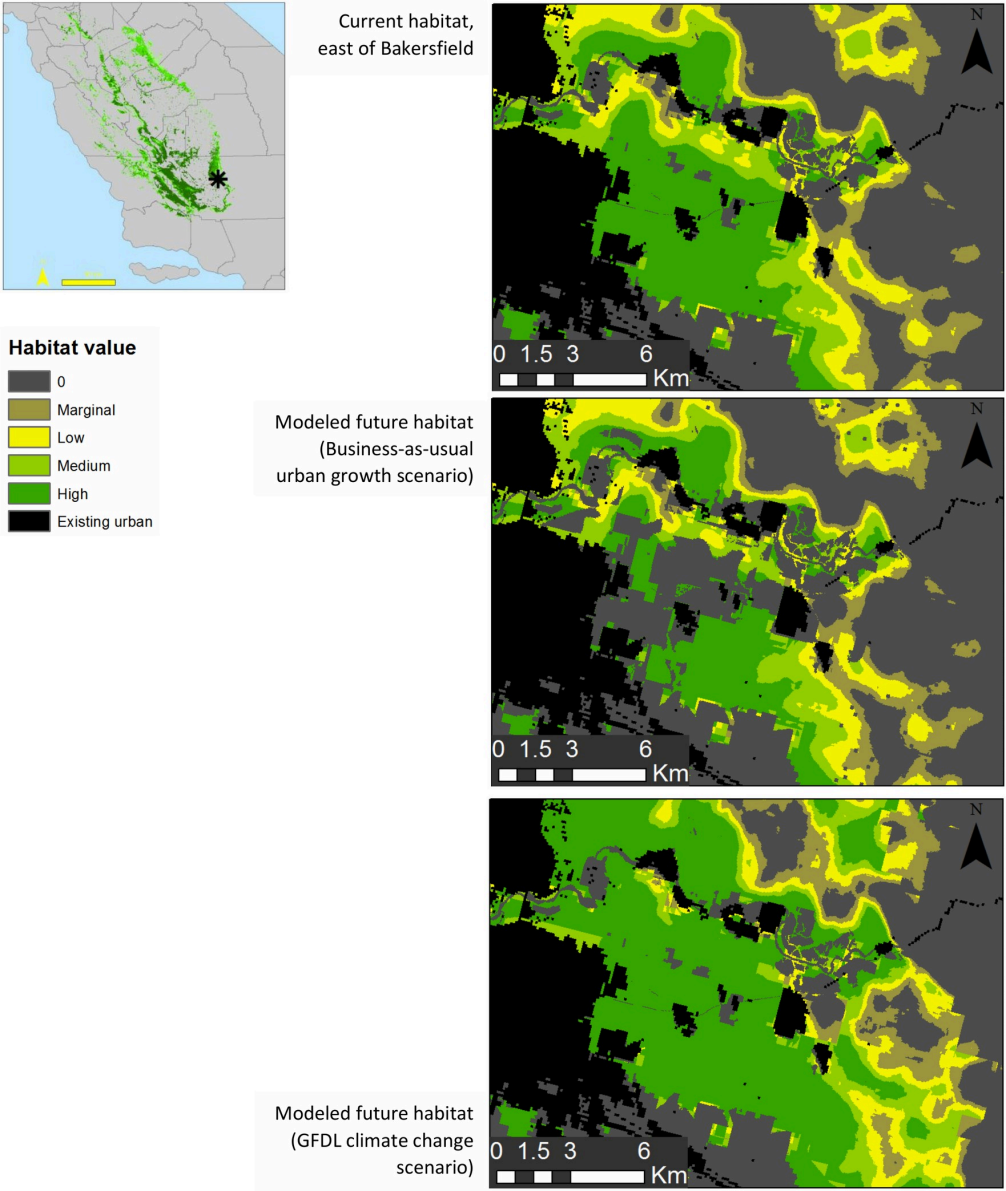
Loss of suitable kit fox habitat in the area to the east of Bakersfield, California under the business-as-usual growth scenario and increase in suitable habitat under the GFDL climate change scenario. Green represents suitable kit fox habitat. Range-wide maps of changes to habitat for both climate scenarios are shown in the S1 Fig. Range-wide maps for changes due to urban growth are not shown because the patchy nature of the grow-out scenarios makes changes difficult to see at the range-wide scale. County outline data are from the National Atlas of the United States. The map was created in ArcMap 10.2.

The business-as-usual land-use change scenario produced a 17% population decline, whereas the compact growth scenario (similar to smart-growth, in which increased development in urban centers is emphasized rather than sprawl outside of existing cities) produced a 15% population decline (Fig 3). As expected, most of this change is around the perimeter of cities and other development. This loss of habitat along the eastern edge of the Central Valley, and at the southern tip of the valley, results in patchier habitat in these regions.

### Impact of climate change

The two scenarios of climate change we considered both resulted in an increase in the quantity of kit fox habitat and a corresponding slight increase in fox population numbers in much of the species’ range (see S1 Table). Both climate models predicted conversion from forest and woodland to grasslands around the perimeter of the San Joaquin Valley. The net increase in grasslands was just over 50% for each model. The CCSM model also predicts a doubling of scrub habitat (see Table 1).

**Table 1:** Quantity of habitat types within the kit fox range for modeled current and future climates.

Land cover type	Modeled current (km^2)	GFDL model (km^2)	% change from current	CCSM model (km^2)	% change from current
Grassland	16,488	25,054	52%	25,909	57%
Blue Oak Woodland	7,453	3,001	-60%	1,811	-76%
Chaparral / Coastal Scrub	4,845	2,779	-43%	860	-82%
Scrub	2,241	2,189	-2%	4,476	100%
Montane Hardwood / Coastal Oak Woodland	1,102	12	-99%	15	-99%
Eastside Pine / Juniper / Pinyon—Juniper	738	129	-82%	92	-88%

Projected vegetation changes also open up a swath of suitable habitat (grassland and scrub vegetation) across two passes separating Bakersfield and Barstow (S2 Fig). Much of this habitat is steep and mountainous, and therefore less suitable as kit fox habitat. Kit foxes generally do not use steep slopes as habitat, but it is unclear whether they use these areas for dispersal. We simulated movement in today’s landscape and in two future, climate-altered landscapes to look for indications of emergent connectivity between the Mojave Desert and the Central Valley fox populations. For this experiment, we substituted male dispersal distances for the female dispersal distances in our model (our original model was a female-only model, and therefore had smaller, female-specific dispersal distances): we allowed foxes to search for a home range along a path with a mean distance of 6.4 km and a standard deviation of 2 km. We ran 100 repetitions of these scenarios over 200 year periods and mapped the routes and frequency of dispersing animals. Simulated kit foxes did indeed disperse between the Mojave Desert and the Central Valley fox populations, although in small numbers and in only one of the two projected future climate scenarios (S2 Fig).

**Fig 4:**
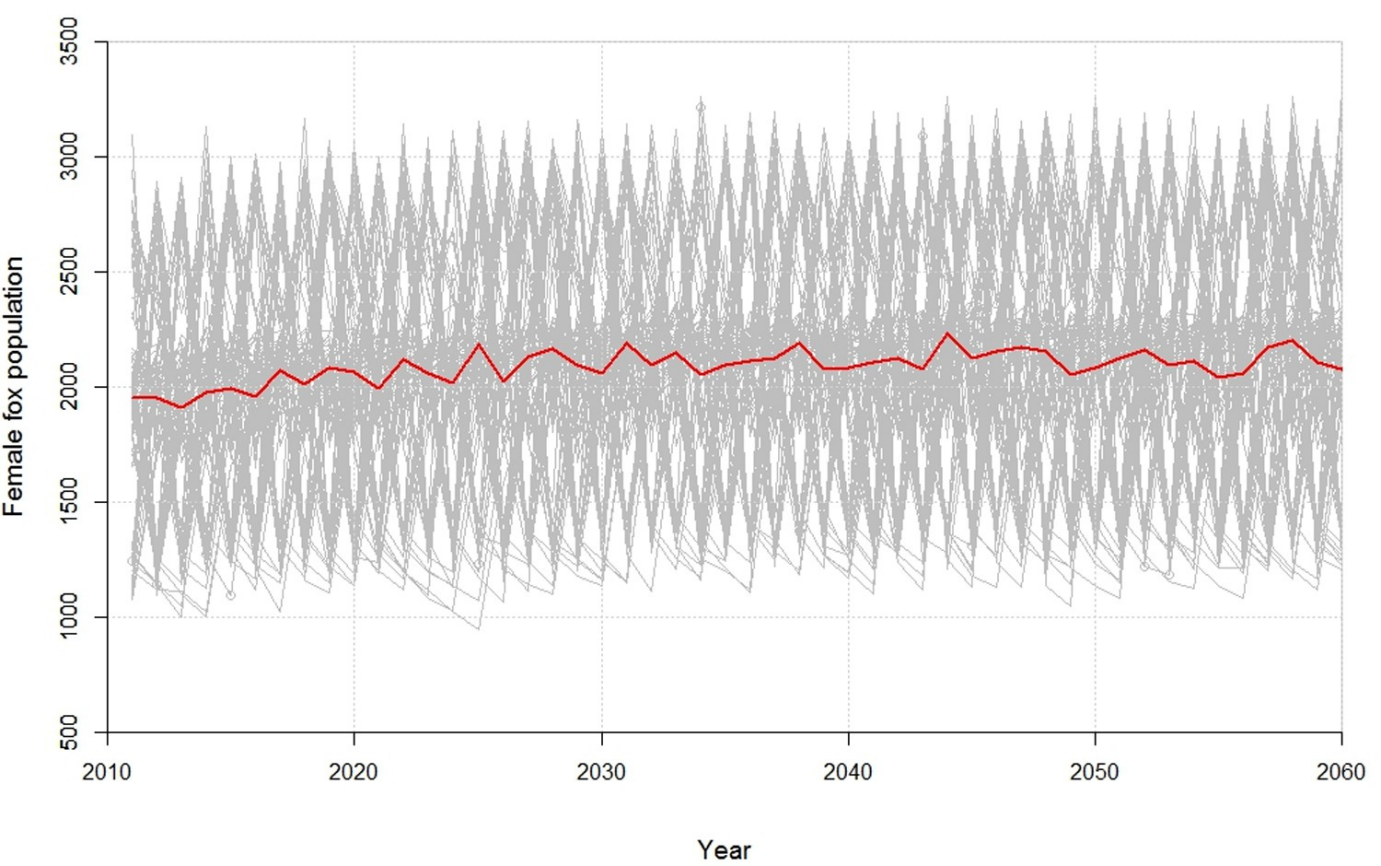
Increased variability in precipitation increases population variance over time, with values fluctuating between 1000 and over 3000 female foxes. The grey lines are the individual iterations and the red line is the mean number of females over 100 iterations.

Meanwhile, increases in climate variability had few impacts on the average population size. As anticipated, increasing climate variability resulted in increased variability in the simulated population trajectories, which in turn raised the probability that the population would fall below a threshold beyond which extinction is increasingly likely (Fig 4).

## Discussion

The endangered San Joaquin kit fox faces pressures from multiple stressors. Projected land-use changes in California’s Central Valley will continue to reduce habitat for this species. Over the same time period, reduced summer precipitation and hotter temperatures can be expected to promote the growth of the grassland and scrub vegetation types that provide habitat for kit foxes. In some parts of the species’ range, such as the area east of Bakersfield (S2 Fig), future habitat loss due to land conversion may be mitigated by climate-change induced range expansion. Thus, the kit fox could be an example of a species that benefits from climate change, though there are multiple reasons why this conclusion could prove overly optimistic (e.g., climate-driven increases in competition or disease).

There are two other principal consequences of climate change that have the potential to negatively impact the kit fox. First, increased variability in rainfall will cause more fluctuations in the kit fox prey, thus decreasing population viability by increasing the likelihood that the population size falls below a critical threshold [22,23]. Also, increasing climate variability and decreasing landscape connectivity can be expected to raise the rate of local demographic extirpations while simultaneously making recolonization increasingly difficult [24].

Second, vegetation changes across the Tehachapi Pass could encourage hybridization between the San Joaquin kit fox and other sub-species of kit fox. No foxes crossed this pass in our current-conditions scenario–a conclusion supported by the literature on kit fox genetics, which suggests that these populations have been separated for some time [25]. Modeled foxes did occasionally cross the pass in one of the climate change scenarios (CCSM), which shows grassland and desert scrub vegetation over the Tehachapi Pass. Although these mountains are steep and are therefore not conducive to use by kit foxes, that may not preclude individuals from occasionally dispersing across the mountains, given suitable vegetation. Hybridization between these subpopulations will have important implications for genetic diversity of kit foxes and for the persistence of kit foxes more generally in the San Joaquin Valley, but it is unclear whether the benefits in terms of persistence of the species outweigh the costs associated with the loss of a subspecies. As shown with our models, increased climate variability could cause extinctions of local San Joaquin kit fox populations within the valley, and dispersing kit foxes from the Mojave Desert could recolonize these habitats.

Further, the solar energy industry is rapidly expanding into the range of the kit fox, and many solar panel installations are planned in areas that currently serve as habitat for the species, with unknown consequences. Solar development was not included in the landuse change models that we used. Instead, we assessed the probable impact of solar farm development by quantifying the amount of overlap between potential solar habitat (from the National Renewable Energy Laboratory) and kit fox habitat. We found that 45.5% of the best habitat (suitability greater than 90%) overlaps with areas designated by areas of potential solar development, but the impacts to kit foxes are unknown.

Although climate change is poised to negatively impact many species of plants and animals around the globe, our results indicate that land-use change and pesticide use will likely have a much stronger negative effect on the San Joaquin kit fox. Indirect effects of climate change on vegetation in the region may actually have positive effects on kit foxes by increasing the extent of their habitat. The degree to which this positive effect is countered by detrimental impacts such as precipitation-driven changes in prey availability is yet to be seen, although our results indicate that the positive impact on vegetation could outweigh at least this one other negative climate impact. A scenario that combines threats and that specifically addresses stressor interactions could be run to assess the impacts on the fox population and determine possible interactions between threats. Our results highlight the importance of considering the impacts that multiple disturbance regimes will have on population dynamics.

## Supporting information

S1 Table UPlan crosswalk table. (PDF)

S1 Fig Figure of climate change impact on kit fox habitat across California. (DOCX)

S2 Fig Possible movement of dispersing individuals between San Joaquin and Mojave populations of kit fox, indicating the possibility of future hybridization. Modeled foxes did not move between the Central Valley and the Mojave Desert in the current scenario or in one of the two projected future scenarios, but a few foxes did cross the path in the CCSM modeled future scenario. Shades of blue represent flux of dispersers across the Tehachapi Pass, with darker blues indicating more foxes moving through those areas. The map was created in ArcMap 10.2.
(TIF)
