# Supplementary material for: Land use change and rodenticide exposure trump climate change as the biggest stressors to San Joaquin kit fox
Source: PLoS One. 2019 Jun 12;14(6):e0214297. doi: 10.1371/journal.pone.0214297 (PMC6561535; doi:10.1371/journal.pone.0214297)
Supplement: S1 Table — (PDF) [file pone.0214297.s001.pdf]

Table S1: UPlan Crosswalk Table.

New suitability values are from Cypher and Phillips (Cypher et al. 2013b) except those with *, which are estimates based on other land use types.

UPlan Land Use Type	Habitat Suitability Value
Commercial High	20*
Industrial	40
Residential 50 (50 units/acre)	10
Residential 20 (20 units/acre)	10
Residential 10 (10 units/acre)	10
Residential 5 (5 units/acre)	10
Commercial Low	40
Residential 1 (1 unit/acre)	10
Residential .5 (2 acre lots)	10
Residential .1 (10 acre lots)	20*
