# Supplementary material for: Land use change and rodenticide exposure trump climate change as the biggest stressors to San Joaquin kit fox
Source: PLoS One. 2019 Jun 12;14(6):e0214297. doi: 10.1371/journal.pone.0214297 (PMC6561535; doi:10.1371/journal.pone.0214297)
Supplement: S1 Fig — (DOCX) [file pone.0214297.s002.docx]

**Figure S1: Figure of climate change impact on habitat**

Current

GFDL projection

CCSM projection

Kit fox habitat

Natural, non-habitat

Developed

Agriculture


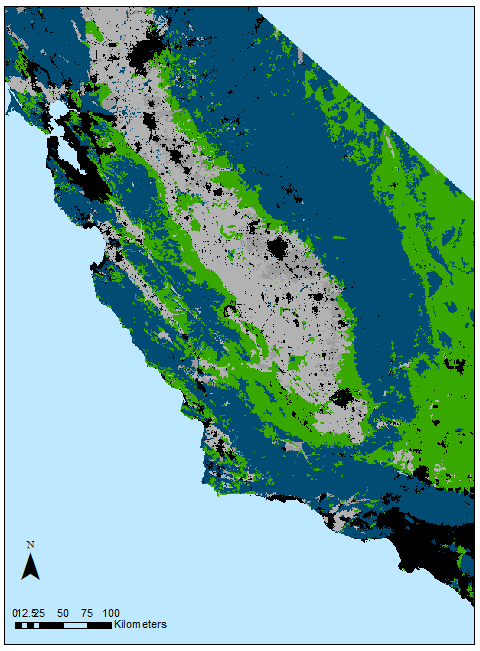

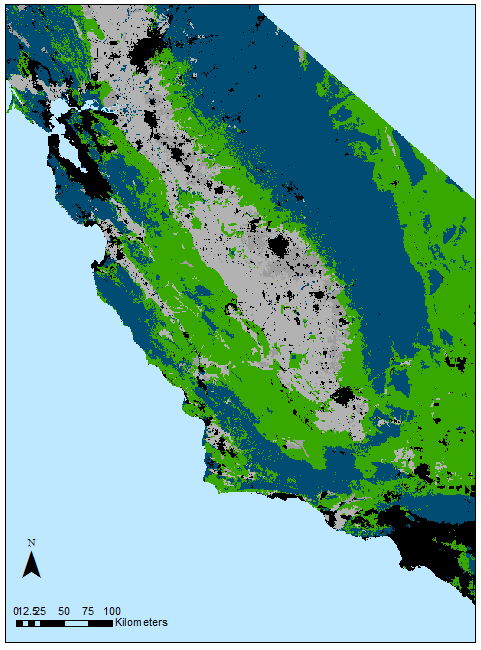

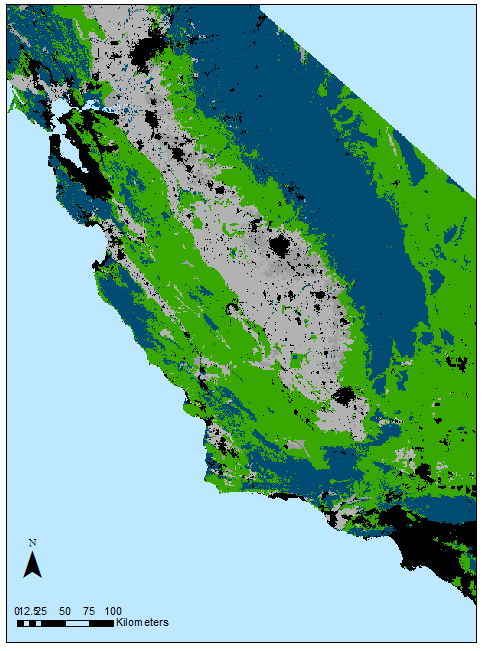


Modeled habitat for San Joaquin kit fox under current climate and two projections (2069). Wide swaths of habitat open up around the perimeter of the San Joaquin Valley in both projections. The map was created in ArcMap 10.2.
