# Supplementary figures and images for: Land use change and rodenticide exposure trump climate change as the biggest stressors to San Joaquin kit fox
Source: PLoS One. 2019 Jun 12;14(6):e0214297. doi: 10.1371/journal.pone.0214297 (PMC6561535; doi:10.1371/journal.pone.0214297)

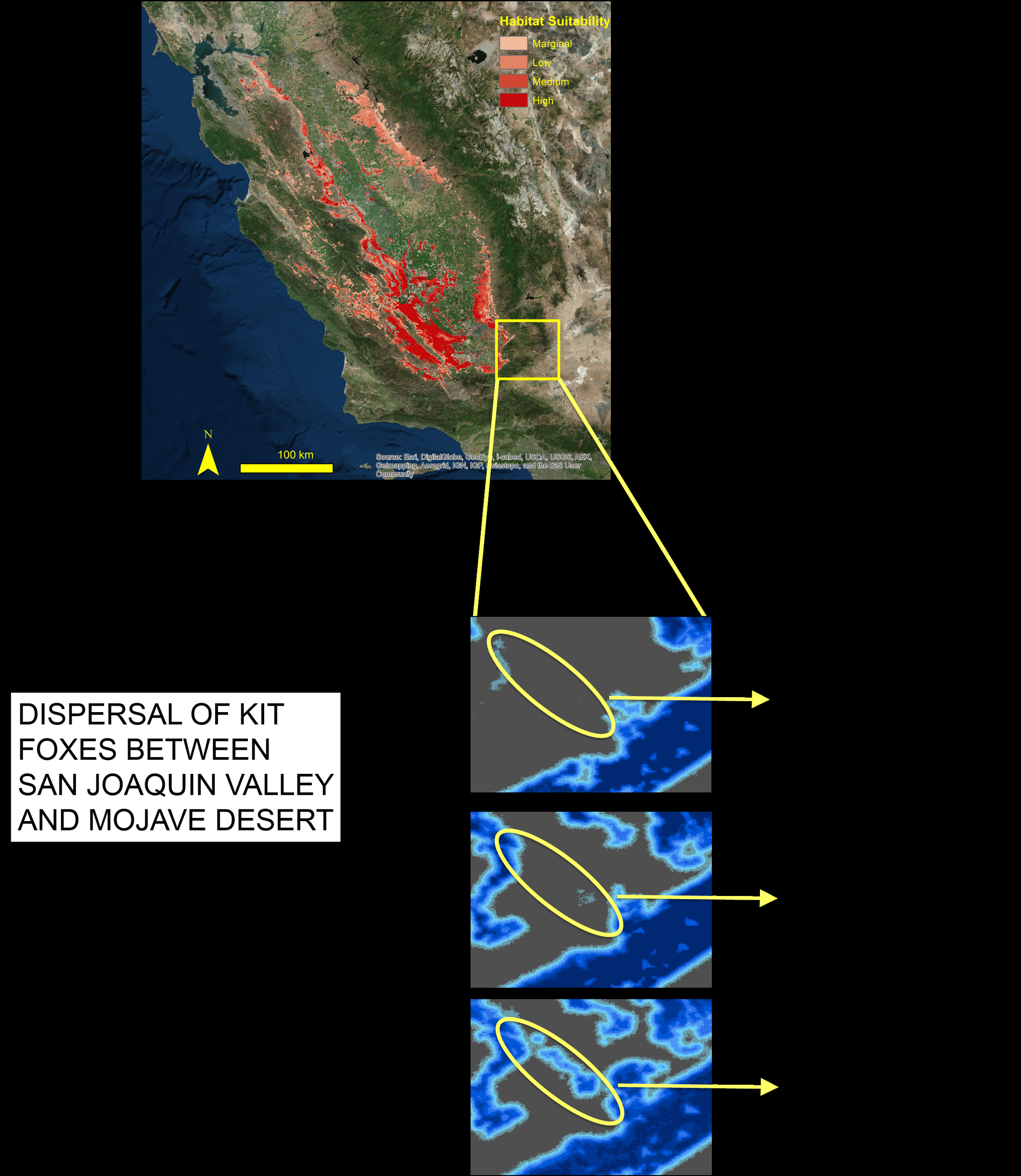

Supplement: S2 Fig — Modeled foxes did not move between the Central Valley and the Mojave Desert in the current scenario or in one of the two projected future scenarios, but a few foxes did cross the path in the CCSM modeled future scenario. Shades of blue represent flux of dispersers across the Tehachapi Pass, with darker blues indicating more foxes moving through those areas. The map was created in ArcMap 10.2. (TIF) [file pone.0214297.s003.tif]
